# P11 Multiple insufficiency fractures in rheumatoid arthritis

**DOI:** 10.1093/rap/rkab068.010

**Published:** 2021-10-19

**Authors:** Qurat Ul Ain Amjad, Spencer Ellis

**Affiliations:** Rheumatology, Lister Hospital, East and North Hertfordshire Trust, Stevenage, United Kingdom

## Abstract

**Case report - Introduction:**

Rheumatoid arthritis (RA) is an autoimmune inflammatory arthropathy with systemic manifestations. It is 4-times more common in females. RA is recognised to induce bone loss and decrease in bone mineral density (BMD). Management may include corticosteroids (CS) for new presentations, acute flares, and more rarely longer-term management, which increases bone fragility. Patients are at 30—50% increased risk of developing osteoporosis with a 30% increase in fracture risk. This risk rises with the level of persistent disease inflammation.

We present a case of a lady with longstanding RA, who sustained multiple bone fractures without significant osteoporosis on bone density scanning.

**Case report - Case description:**

Our patient is a 64-year-old headteacher who took early retirement due to reduced mobility after 20 years of seropositive RA. She had received multiple disease modifying drugs (DMARDs) and biologics therapies, requiring repeated alterations primarily due to treatment failure. She was commenced on alendronic acid due to osteopaenia of the hip but 2 years later sustained a fractured neck of femur and was switched to risedronate.

A year later she presented with acutely painful and swollen right foot and ankle without history of trauma. X-rays showed progressive degenerative change whilst inflammatory markers were normal. Ultrasound demonstrated sub-clinical synovitis. Her medication was optimised but the ankle swelling persisted, rendering her wheelchair-reliant. MRI revealed multiple stress fractures involving calcaneum, talus and 5^th^ proximal phalanx. She was treated with 16 weeks of an Aircast boot. An old right upper medial tibial fracture was also identified.

Repeat dual energy X-ray absorptiometry (DEXA) scan showed osteopaenia but with improvement from the previous scan (T score of -2.1 total hip and -1.6 lumbar vertebra). She smoked 1 cigarette a day, did not drink alcohol and there was no parental history of fractures. No evidence of malabsorption or endocrine disorder was identified. Unusually, she had received tamoxifen in her late 20s for cancer prevention based on breast fibroadenosis and she experienced early menopause aged 36 years.

Inflammatory markers, calcium, parathyroid hormone, and immunoglobulins were normal. Vitamin D3 levels were insufficient at 40.3 nmol/l and replacement was initiated, following which she was switched to intravenous zoledronic acid.

After one infusion of zolendronate, she twisted her right ankle and sustained a new malleolar fracture. She was converted to 6-monthly denosumab injections along with calcium and vitamin D, which has been continued. Her RA remains active, and she has recently commenced JAK2 inhibitors.

**Case report - Discussion:**

Inflammatory arthropathies such as RA predispose to significant morbidity and disability. An earlier age of diagnosis poses a longer inflammatory response in body, with a higher incidence of bone health complications. A treat-to-target strategy in RA aids optimal disease management and reduces fracture risk. Studies have shown the risk of osteoporosis in RA is not just disease dependent but also affected by certain medications.

Treatment challenges arise when a patient sustains fracture despite a BMD above osteoporosis risk criteria, leading us to consider other variables. She was further investigated for secondary causes of osteoporosis, including endocrine causes, and was found to be vitamin D insufficient, which was replaced prior to further antiresorptive treatment.

Our case also highlights a diagnostic dilemma given that our patient presented with a single swollen joint assumed to be due to active RA. Multiple insufficiency fractures were only identified after MRI was performed.

As per EULAR criteria, our patient had difficult to treat RA with a long disease duration. She showed intolerance to a several DMARDs and treatment failure with multiple biologic therapies. She had required local joint injections and repeated short courses of oral steroids. These factors are likely to have played a considerable role in her fracture development. RA is an independent risk factor for fracture in both men and women with disease duration and CS use being important clinical variables.

Bisphosphonates are considered vital in fracture risk reduction. The compliance is an important factor for both primary and secondary prevention of fracture. They are associated with decreased bone remodelling and have been well studied for atypical femoral fractures; however, whether there is any link with stress fractures in the feet requires further studies. Long-term use (>5years) hasn’t shown to be beneficial in preventing hip fractures.

**Case report - Key learning points:**

1. A high DAS28 score is an independent risk factor for developing osteoporosis suggesting higher inflammatory exposure. A treat-to-target strategy should be prioritised as it has shown to reduce bone loss.
2. Osteopaenia in presence of a prior fragility fracture, increases future risks of fracture as would osteoporosis alone. BMD values should not be used in isolation to determine fracture risk and multi-variant assessments, such as FRAX scores, should be undertaken as part of the RA annual review.
3. Dietary vitamin D deficiency should be undertaken in workup of fractures in long-standing RA.
4. Stress fractures should be considered as a cause of unresponsive bone and joint pain in inflammatory arthritis, particularly where other parameters such as inflammatory markers are normal. These are more common in long-standing and persistently active disease.
5. Conventional X-rays may be normal, and MRI is the preferred imaging modality to diagnose stress fractures.
6. Long-term bisphosphonates-related atypical fractures require further studies. Any radiologic screening advised in prolonged exposure to these drugs is not known.
7. Newer osteoporosis medications, including monoclonal antibodies such as denosumab, may provide alternative strategies for the management of bone density reduction related to RA.

